# Supplementary material for: An l-2-hydroxyglutarate biosensor based on specific transcriptional regulator LhgR
Source: Nat Commun. 2021 Jun 15;12:3619. doi: 10.1038/s41467-021-23723-7 (PMC8206213; doi:10.1038/s41467-021-23723-7)
Supplement: Supplementary file 1 — Supplementary Information [file 41467_2021_23723_MOESM1_ESM.pdf]

Supplementary Information

An L-2-hydroxyglutarate biosensor based on specific transcriptional regulator LhgR

Zhaoqi Kang[1], Manman Zhang[2], Kaiyu Gao[1], Wen Zhang[3], Wensi Meng[1], Yidong Liu[1], Dan Xiao[1], Shiting Guo[1], Cuiqing Ma[1], Chao Gao[1,*], Ping Xu[4,*]

[1]*State Key Laboratory of Microbial Technology, Shandong University, Qingdao, People's Republic of China*

[2]*Tianjin Key Laboratory of Radiation Medicine and Molecular Nuclear Medicine, Department of Radiobiology, Institute of Radiation Medicine of Chinese Academy of Medical Science & Peking Union Medical College, Tianjin, People's Republic of China*

[3]*Center for Gene and Immunotherapy, The Second Hospital of Shandong University, Jinan, People's Republic of China*

[4]*State Key Laboratory of Microbial Metabolism, Joint International Research Laboratory of Metabolic & Developmental Sciences, and School of Life Sciences & Biotechnology, Shanghai Jiao Tong University, Shanghai, People's Republic of China*

***Corresponding authors:**

Mailing address for C. Gao: State Key Laboratory of Microbial Technology, Shandong University, Qingdao 266237, People's Republic of China, Tel/Fax: +86-532-58631561, E-mail: jieerbu@sdu.edu.cn.

Mailing address for P. Xu: State Key Laboratory of Microbial Metabolism, and School of Life Sciences & Biotechnology, Shanghai Jiao Tong University, Shanghai 200240, People's Republic of China, Tel/Fax: +86-21-34206723, E-mail: pingxu@sjtu.edu.cn.

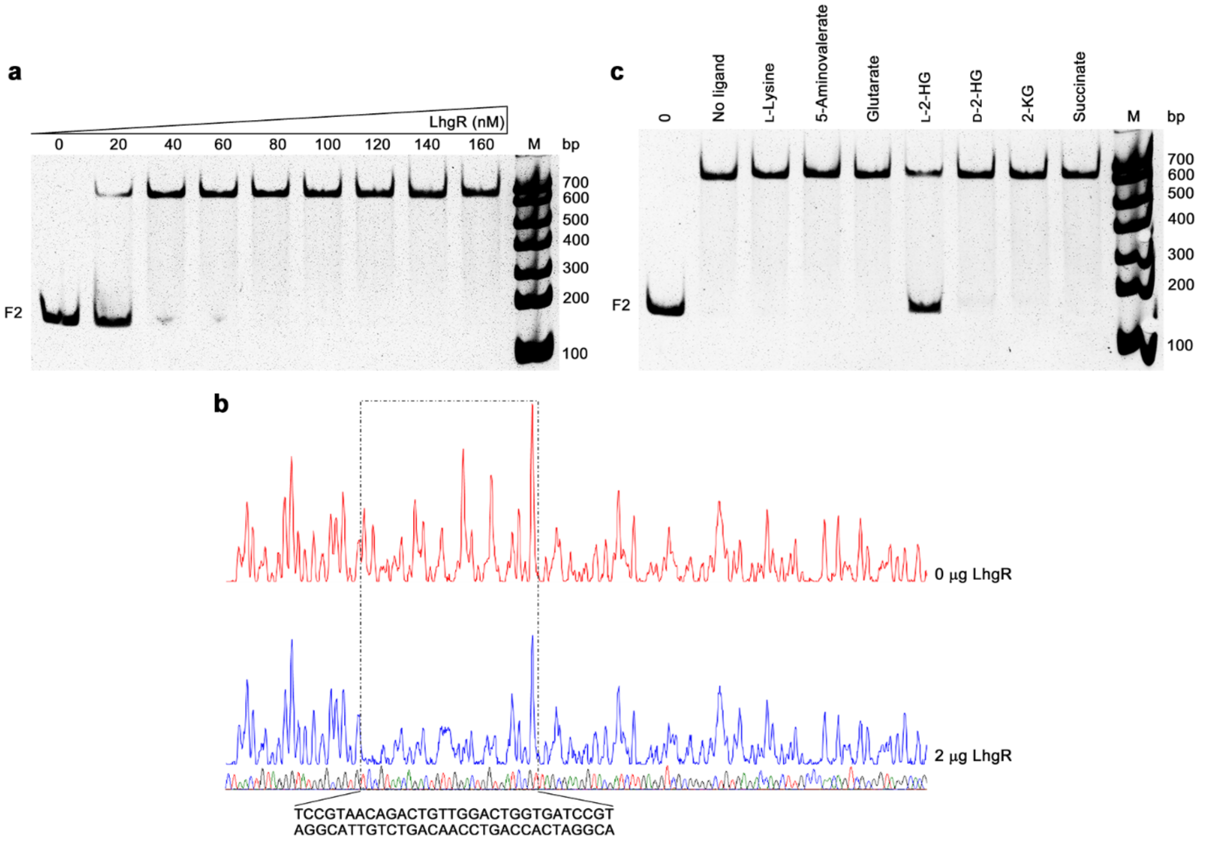

Supplementary Figure 1 Analysis of the interaction between LhgR and the *lhgR* promoter region. **(a)** LhgR can bind to the *lhgR* promoter region. F2 fragment containing the *lhgR* promoter region (10 nM) was titrated by purified LhgR (0, 20, 40, 60, 80, 100, 120, 140, 160 nM). Lane M, molecular weight markers. **(b)** DNase I footprinting analysis of LhgR binding to the *lhgR* promoter region. The F2 fragment was labeled with 6-carboxyfluorescein (FAM) and incubated with 2 μg LhgR (blue line) or without LhgR (red line). The region protected by LhgR is indicated with a dotted box. **(c)** L-2-HG prevent LhgR binding to the *lhgR* promoter region. EMSAs were carried out with F2 fragment (10 nM) and purified LhgR (70 nM) in the absence of any other tested compounds (No ligand) and in the presence of 30 mM different

compounds. Lane M was the molecular weight markers; lane 0 without LhgR was used as the control. Source data are provided as a Source Data file.

MVSKGEETTMGVIKPDMKIKLKMEGNVNGHAFVIEGEGEGKPYDGTNTINLE
VKEGAPLPFSYDILTTAFAYGNRAFTKYPDDIPNYFKQSFPEGYSWERTMTF
EDKGIVKVKSDISMEEDSFIYEIHLKGENFPPNGPVMQKKTTGWDASTERMY
VRDGVLKGDVKHKLLLEGGGHHRVDFKTIYRAKKAVKLPDYHFVDHRIEILN
HDKDYNKVTVYESAVARNSTDGMDELYKELMLELQRPDTLVERVVSAIRAEI
DSGRLAAEARLPTEQQLAEQLNVSRSVVREAVAQLKADGVLIARRGLGSYIS
KTPGGTVFRFPGSTGRKPDLVQMFEMRLWIETQAAAIAARRRDEHDLANMA
QALQEMLDKRSDFATASAADVAFHRAIAEASKNDYFVAFHDFLGGQLANAR
RTAWENSAAHSVGGSAEANREHQALYQAIADGDRQRAAACAEAHLRASAK
RLKIELPALDVDMVSKGEELFTGVVPILVELDGDVNGHKFSVSGEGEGDAT
YGKLTLKLICTTGKLPVPWPTLVTTLGYGLQCFARYPDHMKQHDFFKSAMP
EGYVQERTIFFKDDGNYKTRAEVKFEGDTLVNRIELKGIDFKEDGNILGHKLE
YNYNSHNVYITADKQKNGIKANFKIRHNIEDGGVQLADHYQQNTPIGDGPVL
LPDNHYLSYQSALSKDPNEKRDHMVLLEFVTAAGITLGMDELYK*

Supplementary Figure 2 Full protein sequence of LHGFR$_{0N0C}$. The sequence of mTFP, LhgR, and Venus are highlighted by cyan, gray, and yellow, respectively. Truncation sites are indicated with dotted boxes. EL and VD are the amino acid sequences of the restriction sites SacI and SalI, respectively, and indicated with underlines.

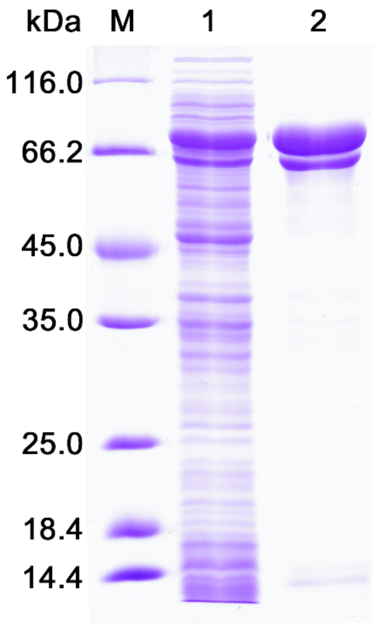

Supplementary Figure 3 SDS-PAGE analysis of the purification of LHGFR$_{0N0C}$. Lane M, molecular weight markers; lane 1, crude extract of *E. coli* BL21(DE3) harboring pETDuet-LHGFR$_{0N0C}$; lane 2, purified His$_6$-tagged LHGFR$_{0N0C}$ using a HisTrap column. Source data are provided as a Source Data file.

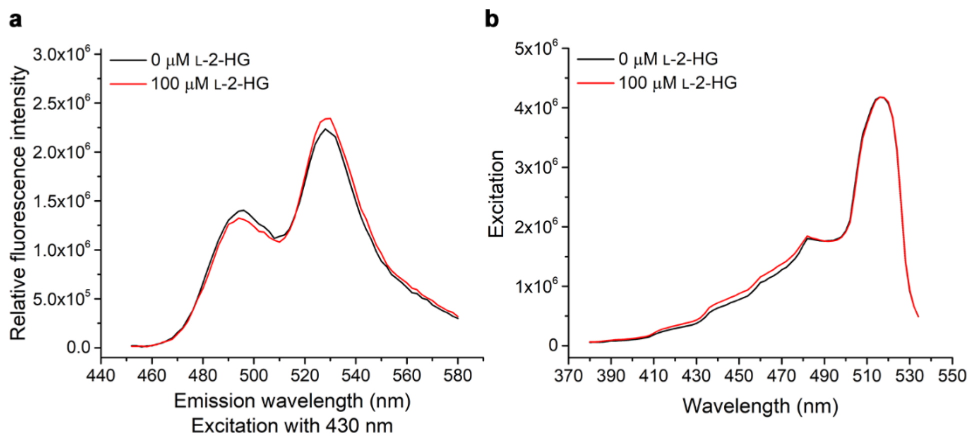

Supplementary Figure 4 Spectra properties of LHGFR0N0C. **(a)** Fluorescence emission spectrum changes of 1 µM LHGFR0N0C at 430 nm excitation with (red) or without (black) the addition of 100 µM L-2-HG were indicated. **(b)** Fluorescence excitation spectrum changes of 1 µM LHGFR0N0C with (red) or without (black) the addition of 100 µM L-2-HG were indicated, emission was measured at 550 nm with excitation from 380 to 535 nm in steps of 2 nm. Source data are provided as a Source Data file.

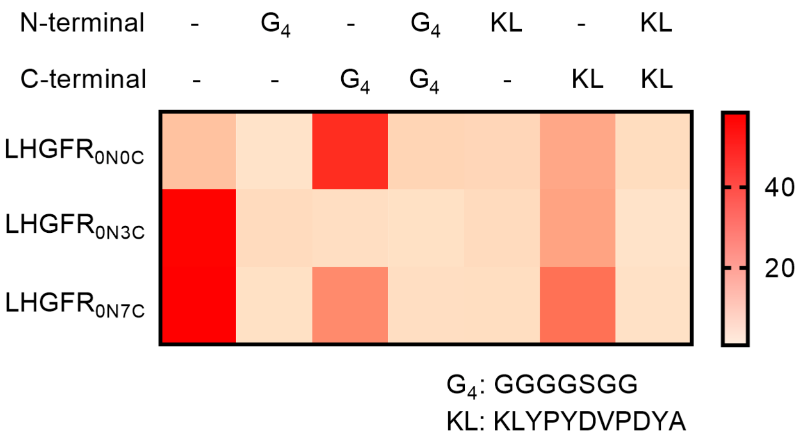

Supplementary Figure 5 Heap map of ΔR_{max} to the LHGFR variants in which a series of artificial linkers were added between LhgR and fluorescent proteins. G_4 and KL indicate the flexible linker Gly-Gly-Gly-Gly-Ser-Gly-Gly and rigid linker Lys-Leu-Tyr-Pro-Tyr-Asp-Val-Pro-Asp-Tyr-Ala, respectively. Color indicates the value of ΔR_{max}. Source data are provided as a Source Data file.

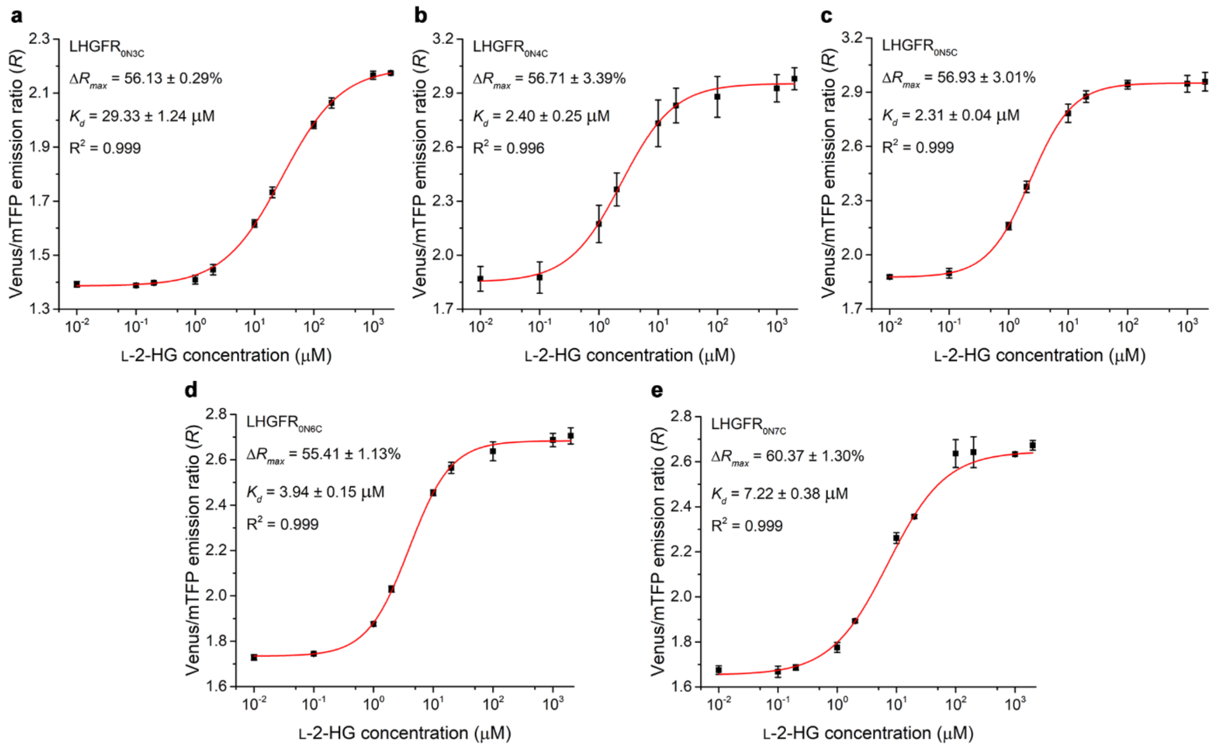

Supplementary Figure 6 Dose-response curves of purified LHGFR variants truncated three to seven C-terminal amino acids in LhgR for L-2-HG. Dose-response curves of LHGFR$_{0N3C}$ **(a)**, LHGFR$_{0N4C}$ **(b)**, LHGFR$_{0N5C}$ **(c)**, LHGFR$_{0N6C}$ **(d)**, and LHGFR$_{0N7C}$ **(e)** for increasing concentrations (10 nM to 2 mM) of L-2-HG were indicated. The maximum ratio change (ΔR_{max}), apparent dissociation constant (K_d), and R^2 were shown in each figure. All data shown are means ± s.d. (n = 3 independent experiments). Source data are provided as a Source Data file.

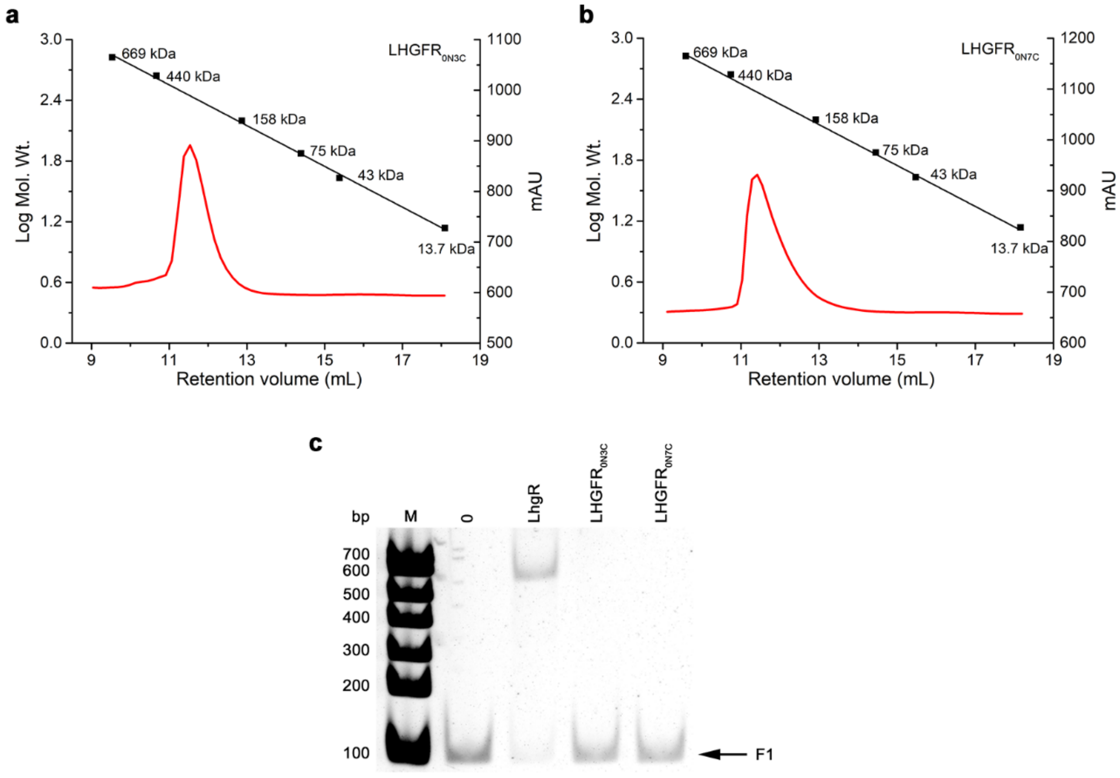

Supplementary Figure 7 Analysis of the native molecular weight and DNA binding ability of LHGFR$_{0N3C}$ and LHGFR$_{0N7C}$. **(a, b)** Gel-filtration chromatography of the purified LHGFR$_{0N3C}$ **(a)** and LHGFR$_{0N7C}$ **(b)** with the Superdex 200 10/300 GL column. Red curve, chromatogram of purified LHGFR; Black line, standard curve for protein molecular mass standards. **(c)** F1 fragment containing the *lhgO* promoter region (10 nM) was titrated by purified LhgR (60 nM), LHGFR$_{0N3C}$ (60 nM), and LHGFR$_{0N7C}$ (60 nM) in EMSAs, respectively. LhgR could bind to the *lhgO* promoter region, while LHGFR$_{0N3C}$ and LHGFR$_{0N7C}$ could not. Lane M was the molecular weight markers; lane 0 with only F1 fragment was used as the control. Source data are provided as a Source Data file.

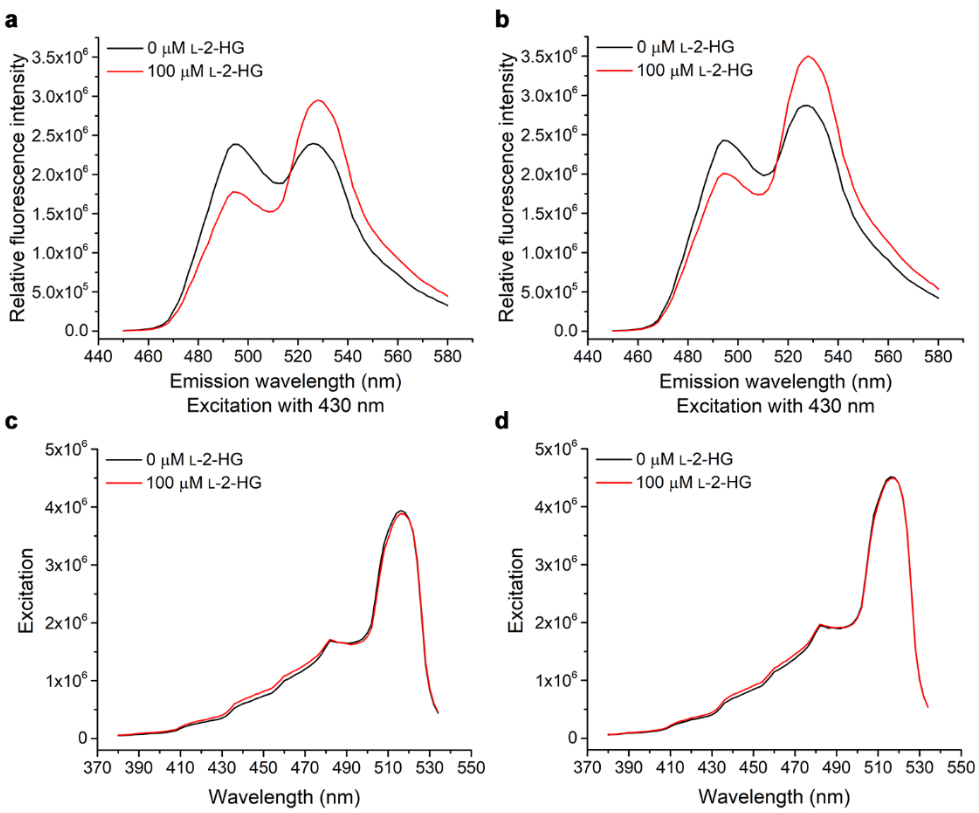

Supplementary Figure 8 Spectra properties of LHGFR$_{0N3C}$ and LHGFR$_{0N7C}$. **(a, b)** Fluorescence emission spectra changes of 1 μM LHGFR$_{0N3C}$ **(a)** and LHGFR$_{0N7C}$ **(b)** at 430 nm excitation with (red) or without (black) the addition of 100 μM L-2-HG were indicated. **(c, d)** Fluorescence excitation spectra changes of 1 μM LHGFR$_{0N3C}$ **(c)** and LHGFR$_{0N7C}$ **(d)** with (red) or without (black) the addition of 100 μM L-2-HG were indicated, emission was measured at 550 nm with excitation from 380 to 535 nm in steps of 2 nm. Source data are provided as a Source Data file.

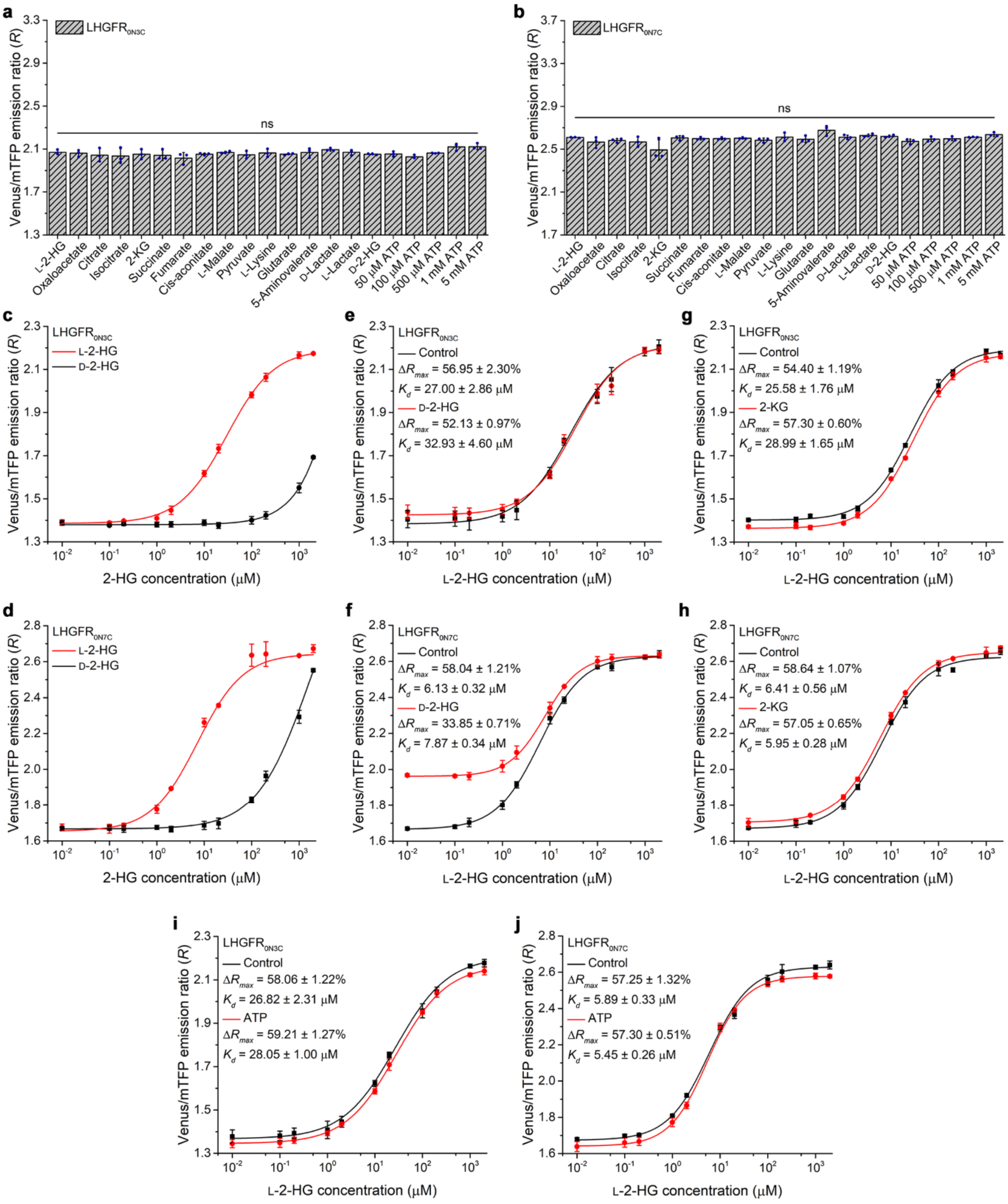

Supplementary Figure 9 Influence of various metabolites on detection of L-2-HG by LHGFR$_{0N3C}$ and LHGFR$_{0N7C}$. **(a, b)** The effect of 240 μM D-lactate, L-lactate, D-2-HG, different intermediates of TCA cycle and L-lysine catabolism, or different concentrations of

ATP on the detection of L-2-HG by $LHGFR_{0N3C}$ **(a)** and $LHGFR_{0N7C}$ **(b)**. The leftmost column in the presence of only L-2-HG was used as the control. **(c, d)** Comparison of the binding of L-2-HG and its mirror-image enantiomer D-2-HG with $LHGFR_{0N3C}$ **(c)** and $LHGFR_{0N7C}$ **(d)**. Dose-response curves of purified LHGFR for increasing concentrations (10 nM to 2 mM) of L-2-HG and D-2-HG were indicated. The dose-response curve of purified $LHGFR_{0N3C}$ for D-2-HG could not be fitted but the apparent K_d in response to D-2-HG was obviously on the order of millimoles. Purified $LHGFR_{0N7C}$ displayed a higher apparent K_d in response to D-2-HG (1.85 ± 0.91 mM; black line) than L-2-HG (7.22 ± 0.38 μM; red line). **(e-j)** Dose-response curves of $LHGFR_{0N3C}$ **(e, g, and i)** and $LHGFR_{0N7C}$ **(f, h, and j)** for increasing concentrations (10 nM to 2 mM) of L-2-HG were measured in the presence of 240 μM D-2-HG **(e, f)**, 1 mM 2-KG **(g, h)**, or 1 mM ATP **(i, j)**. All data shown are means ± s.d. (n = 3 independent experiments). ns, no significant difference ($P \geq 0.05$) in two-tailed t test. Source data are provided as a Source Data file.

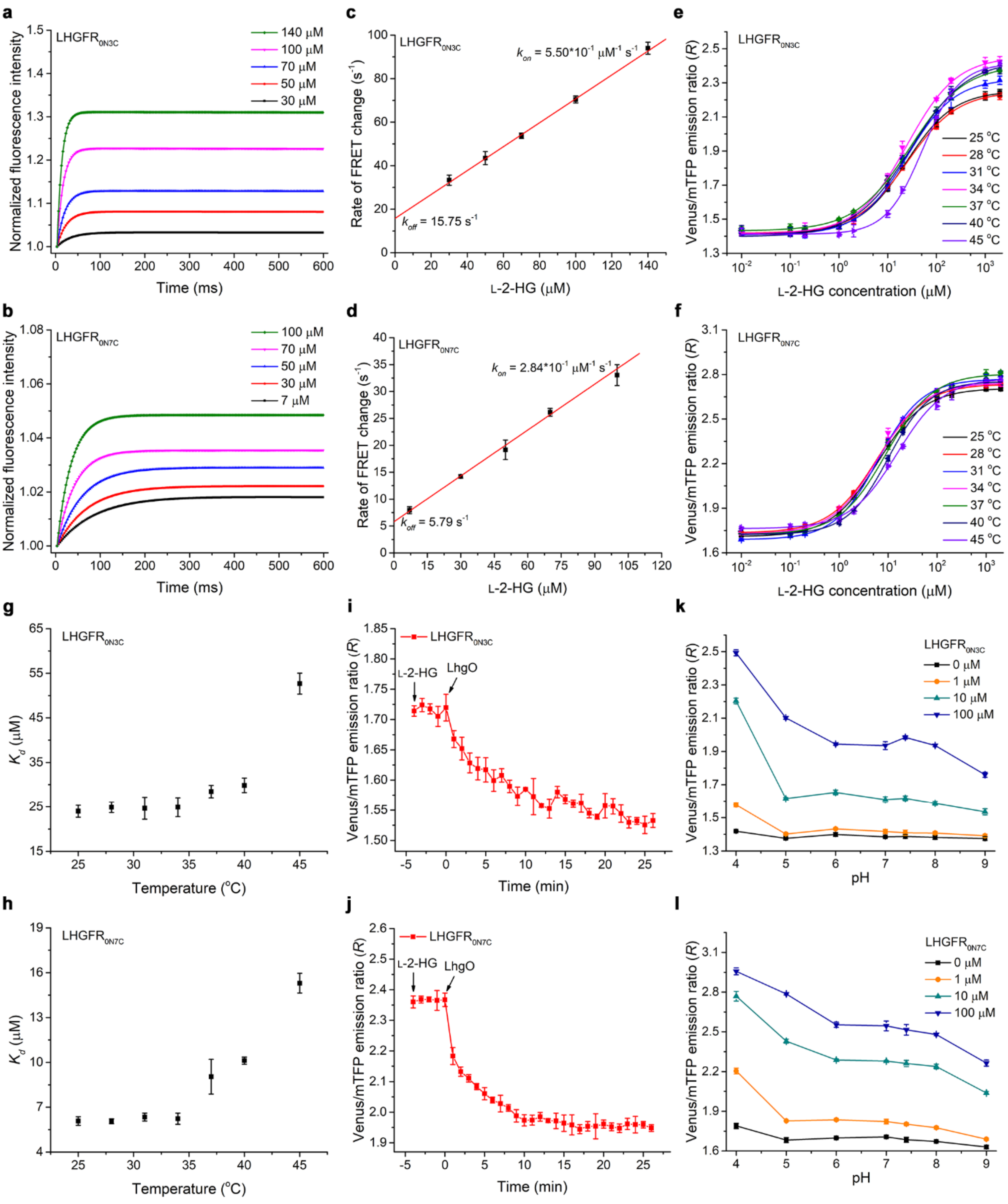

Supplementary Figure 10 *In vitro* characterization of purified LHGFR0N3C and LHGFR0N7C. **(a, b)** Kinetics of L-2-HG binding to LHGFR0N3C **(a)** and LHGFR0N7C **(b)** were measured by using the stopped-flow technique. Different L-2-HG concentrations were mixed with the purified LHGFR0N3C **(a)** and LHGFR0N7C **(b)** protein and the change in Venus fluorescence

intensity was measured over time. The Venus fluorescence intensity change fitted by a single exponential equation corresponding to each L-2-HG concentration was shown. **(c, d)** Apparent rate constants ($k^{app} = k_{on}$[L-2-HG] + k_{off}) determined by fitting the Venus fluorescence increase after L-2-HG addition with a single exponential equation were plotted against L-2-HG concentrations ([L-2-HG]). **(e, f)** Dose-response curves of LHGFR$_{0N3C}$ **(e)** and LHGFR$_{0N7C}$ **(f)** for increasing concentrations (10 nM to 2 mM) of L-2-HG were measured at varying temperatures, ranging from 25 ºC to 45 ºC. **(g, h)** Determined apparent K_d values of LHGFR$_{0N3C}$ **(g)** and LHGFR$_{0N7C}$ **(h)** were plotted against temperatures (the K_d values were determined from **e** and **f**). **(i, j)** Time course of emission ratio changes of LHGFR$_{0N3C}$ **(i)** and LHGFR$_{0N7C}$ **(j)** to sequential addition of 20 μM L-2-HG and 5 μM purified LhgO. The conversion of L-2-HG to 2-KG catalyzed by LhgO could reverse the binding of L-2-HG to LHGFR. 5 μM purified LhgO was added at time point zero (min). **(k, l)** pH-stability analysis of purified LHGFR$_{0N3C}$ **(k)** and LHGFR$_{0N7C}$ **(l)**. Emission ratios of both biosensors in the presence of L-2-HG (0, 1, 10, and 100 μM) were determined at the indicated pH values. All data shown are means ± s.d. (n = 3 independent experiments). Source data are provided as a Source Data file.

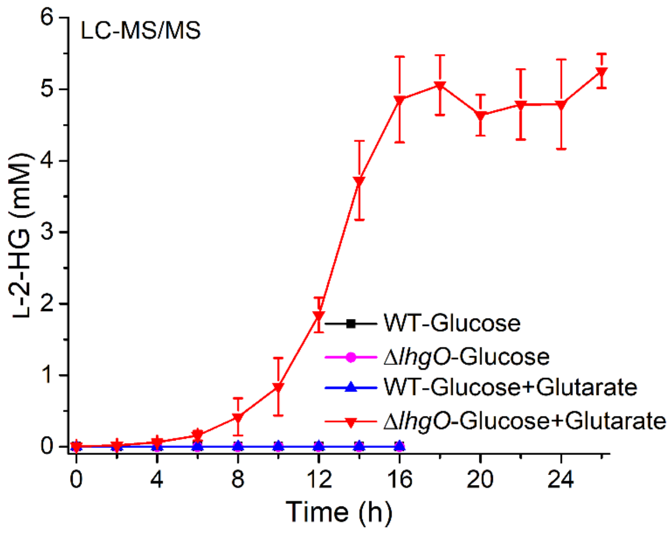

Supplementary Figure 11 Determination of extracellular L-2-HG accumulation of *P. putida* KT2440 and its *lhgO* mutant cultured in 20 mM glucose and 5 mM glutarate as the carbon sources by LC-MS/MS. All data shown are means ± s.d. (n = 3 independent experiments). Source data are provided as a Source Data file.

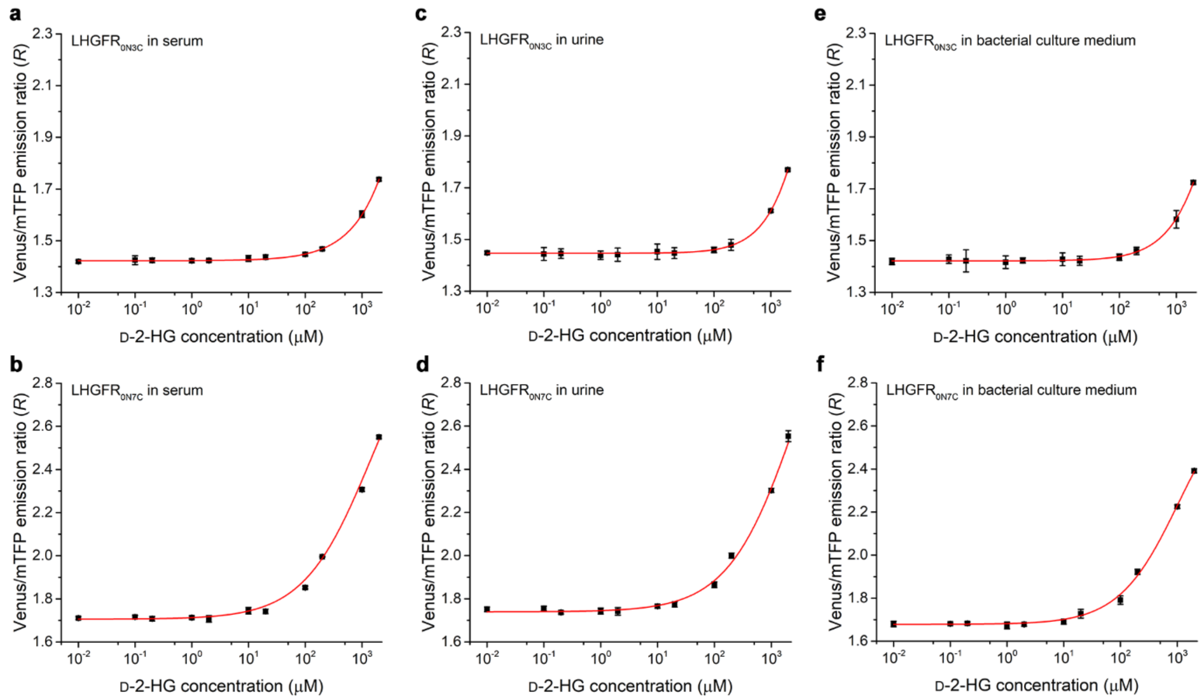

Supplementary Figure 12 Responses of purified LHGFR to D-2-HG in body fluids and bacterial culture system. Dose-response curves of purified LHGFR0N3C **(a, c, and e)** and LHGFR0N7C **(b, d, and f)** for increasing concentrations (10 nM to 2 mM) of D-2-HG in serum **(a, b)**, urine **(c, d)**, and bacterial culture medium **(e, f)**. All data shown are means ± s.d. (n = 3 independent experiments). Source data are provided as a Source Data file.

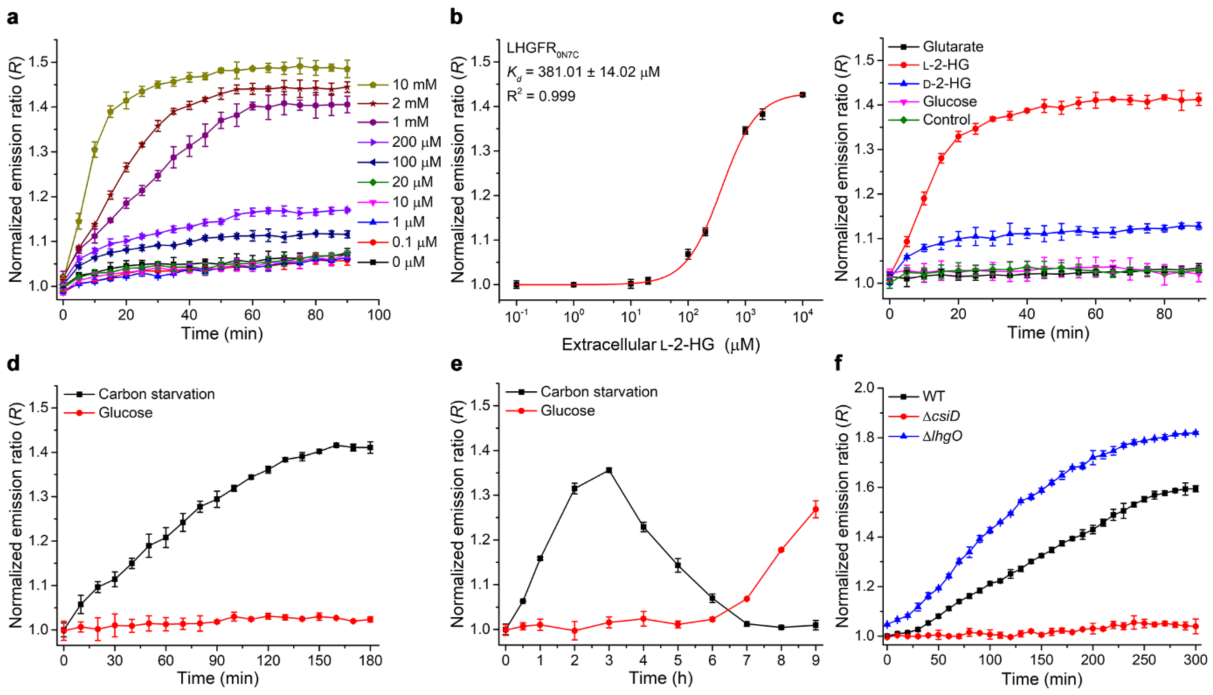

Supplementary Figure 13 Monitoring L-2-HG fluctuations in living bacteria by LHGFR0N7C. **(a)** Time course of the emission ratio changes of LHGFR0N7C expressed in *E. coli* BL21(DE3) in response to exogenous L-2-HG addition. All ratios were normalized to the control (ratio in the absence of L-2-HG at time point zero). **(b)** Normalized dose-response curve of LHGFR0N7C expressed in *E. coli* BL21(DE3) for increasing concentrations (100 nM to 10 mM) of L-2-HG at time point 60 min. **(c)** Time course of the emission ratio changes of LHGFR0N7C expressed in *E. coli* BL21(DE3) in response to the addition of 1 mM glutarate, L-2-HG, D-2-HG, or glucose. All data were normalized to the control (ratio in the absence of any tested compounds at time point zero). **(d)** Detection of carbon starvation-induced L-2-HG accumulation over time by LHGFR0N7C expressed in *E. coli* BL21(DE3). Emission ratio changes of LHGFR0N7C were measured when cultured in carbon starvation medium (black line) and medium with 20 mM glucose (red line). All data were normalized to samples under

carbon starvation condition at time point zero. **(e)** Long-term detection of L-2-HG fluctuations by $LHGFR_{0N7C}$ expressed in *E. coli* BL21(DE3). All data were normalized to samples under carbon starvation condition at time point zero. **(f)** Identification of the roles of CsiD and LhgO in endogenous L-2-HG catabolism during carbon starvation by $LHGFR_{0N7C}$. Emission ratio changes of $LHGFR_{0N7C}$ expressed in *E. coli* MG1655(DE3) wild-type (black line), *E. coli* MG1655(DE3) (Δ*csiD*) (red line), and *E. coli* MG1655(DE3) (Δ*lhgO*) (blue line) were measured in carbon starvation medium. Emission ratios were normalized to time point zero of wild-type strain. All data shown are means ± s.d. (n = 3 independent experiments). Source data are provided as a Source Data file.

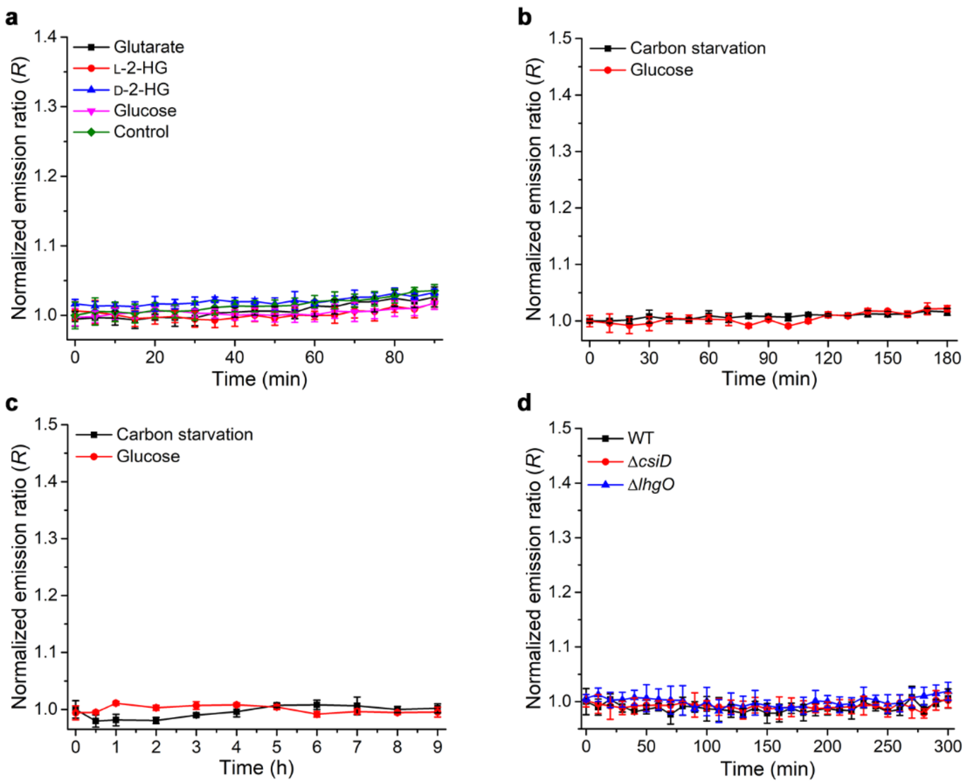

Supplementary Figure 14 Monitoring L-2-HG fluctuations in living bacteria by L-2-HG-insensitive variant LHGFR$_{3N7C}$. **(a)** Time course of the emission ratio of LHGFR$_{3N7C}$ expressed in *E. coli* BL21(DE3) in response to the addition of 1 mM glutarate, L-2-HG, D-2-HG, or glucose. All data were normalized to the control (ratio in the absence of any tested compounds at time point zero). **(b)** Detection of carbon starvation-induced L-2-HG accumulation over time by LHGFR$_{3N7C}$ expressed in *E. coli* BL21(DE3). All data were normalized to samples under carbon starvation condition at time point zero. **(c)** Long-term detection of L-2-HG by LHGFR$_{3N7C}$ expressed in *E. coli* BL21(DE3). All data were normalized to samples under carbon starvation condition at time point zero. **(d)** Identification of the roles of CsiD and LhgO in endogenous L-2-HG catabolism during carbon starvation by LHGFR$_{3N7C}$. Emission ratio changes of LHGFR$_{3N7C}$ expressed in *E. coli* MG1655(DE3)

wild-type (black line), *csiD* mutation (red line), and *lhgO* mutation (blue line) were measured in carbon starvation medium. Emission ratios were normalized to time point zero of wild-type strain. All data shown are means ± s.d. (n = 3 independent experiments). Source data are provided as a Source Data file.

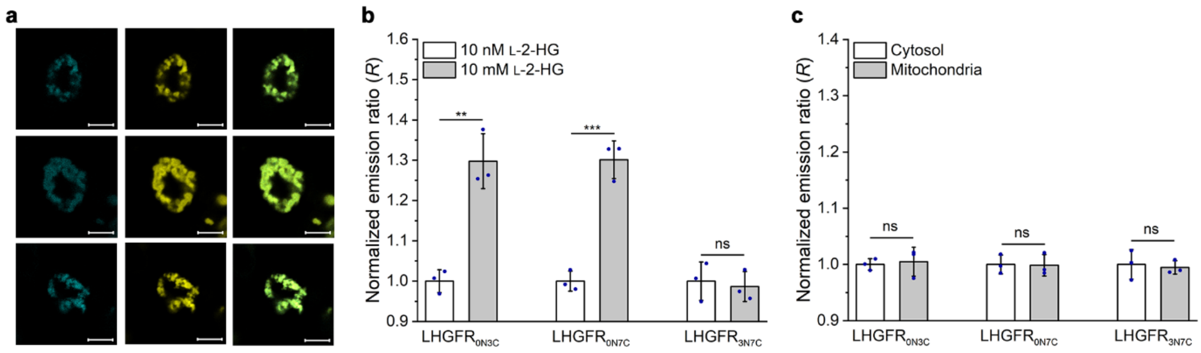

Supplementary Figure 15 Mitochondrial localized LHGFR. **(a)** Confocal microscopy images of mitochondrial $LHGFR_{0N3C}$ (top), $LHGFR_{0N7C}$ (middle), and $LHGFR_{3N7C}$ (bottom)-expressing HEK293FT cells. The images are represented as mTFP channel, Venus channel, and overlay channel from left to right. Scale bar, 10 μm. **(b)** Emission ratio changes of mitochondrial $LHGFR_{0N3C}$, $LHGFR_{0N7C}$, and $LHGFR_{3N7C}$ expressed in HEK293FT cells in respond to 10 nM L-2-HG and 10 mM L-2-HG. Mitochondrial LHGFR-expressing HEK293FT cells were permeabilized with 10 μM digitonin, then 10 nM and 10 mM L-2-HG were added into the treated cell suspension, respectively, and the emission ratios were recorded. Mitochondrial $LHGFR_{0N3C}$ and $LHGFR_{0N7C}$ could respond to L-2-HG application, while $LHGFR_{3N7C}$ could not. The emission ratios were normalized to samples in the presence of 10 nM L-2-HG. P = 0.0022, 0.0006, 0.7232 (from left to right). **(c)** Comparison of L-2-HG concentrations between cytosol and mitochondria. The emission ratios of LHGFR localized in cytosol and mitochondria were recorded, respectively, and there was no detectable difference in emission ratio between cytosol and mitochondrial. The emission ratios were normalized to cells expressed cytosolic LHGFR. All data shown are means ± s.d. (n = 3 independent experiments). **, $P < 0.01$ in two-tailed t test; ***, $P < 0.001$ in two-tailed t test;

ns, no significant difference ($P \geq 0.05$) in two-tailed *t* test. Source data are provided as a Source Data file.

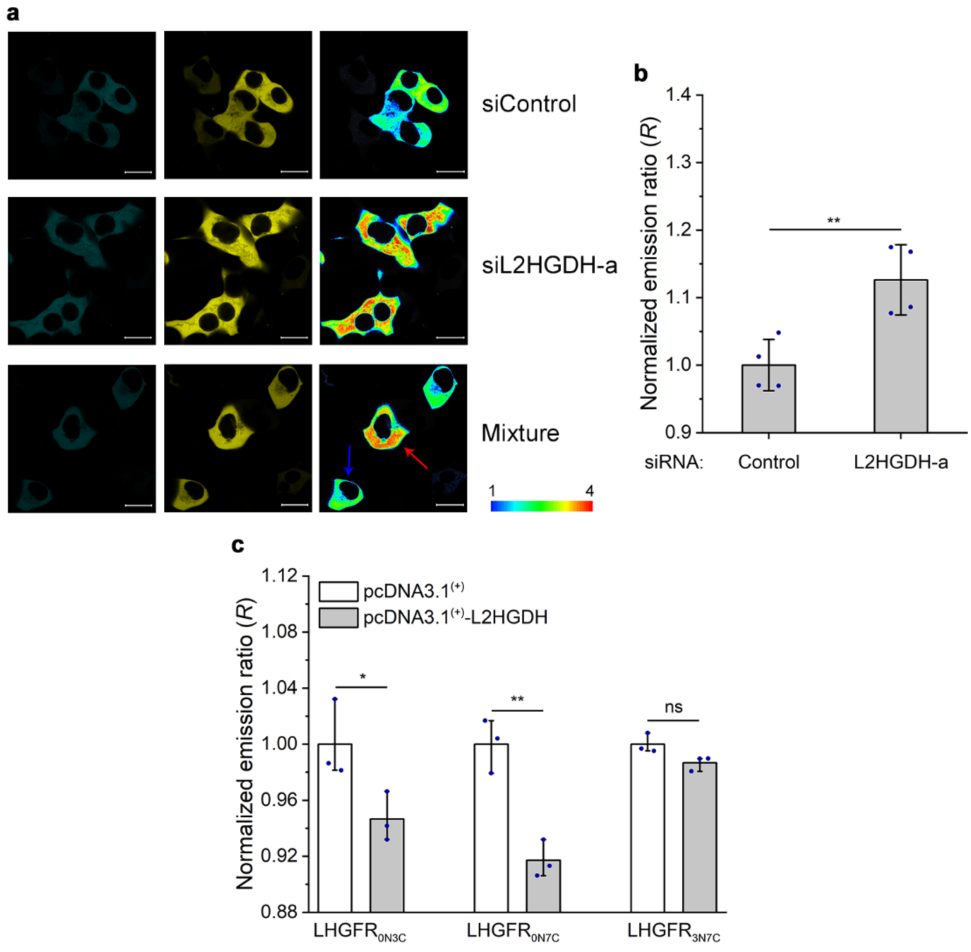

Supplementary Figure 16 Functions of L2HGDH in endogenous L-2-HG catabolism of HEK293FT cells. **(a)** Confocal microscopy images of HEK293FT cells with altered L-2-HG metabolism. HEK293FT cells were co-transfected with LHGFR$_{0N3C}$ and siControl or siL2HGDH-a. Cells treated with different siRNAs were trypsinized separately 24 h following transfection to prepare cell suspensions with or without L2HGDH knockdown. The two types of cells, as well as their mixture with a ratio of 1:1, were re-plated on poly-L-lysine pre-coated 35 mm glass bottom dishes, respectively. Then, live-cell imaging was carried out 24 h following plating. The images are represented as LHGFR$_{0N3C}$-expressing HEK293FT with siControl (top), LHGFR$_{0N3C}$-expressing HEK293FT with siL2HGDH-a (middle), and their

mixture (bottom); mTFP channel (left), Venus channel (middle), and Venus/mTFP emission ratio (right, pseudocolored). The emission ratio of the cell indicated by red arrow was calculated to be 15.10% higher than that of the cell indicated by blue arrow (from the bottom-right image). Scale bar, 20 μm. **(b)** Emission ratio of the HEK293FT cells inside the top image and middle image of **(a)**. Knockdown of L2HGDH induced approximately 12.62% increase in the emission ratio of HEK293FT cells. The data shown are means ± s.d. (n = 4 cells) and normalized to the HEK293FT cells without L2HGDH knockdown. *P* = 0.0078. **(c)** Emission ratio of LHGFR-expressing HEK293FT cells with or without L2HGDH overexpression. HEK293FT cells were co-transfected with LHGFR and empty plasmid pcDNA3.1$^{(+)}$ or pcDNA3.1$^{(+)}$-L2HGDH, and the emission ratios were recorded 48 h following transfection. The emission ratios of $LHGFR_{0N3C}$ and $LHGFR_{0N7C}$ decreased in respond to L2HGDH overexpression, while $LHGFR_{3N7C}$ did not. The data shown are means ± s.d. (n = 3 independent experiments) and normalized to the HEK293FT cells without L2HGDH overexpression. *P* = 0.0494, 0.0035, 0.0570 (from left to right). *, $P < 0.05$ in two-tailed *t* test; **, $P < 0.01$ in two-tailed *t* test; ns, no significant difference ($P \geq 0.05$) in two-tailed *t* test. Source data are provided as a Source Data file.

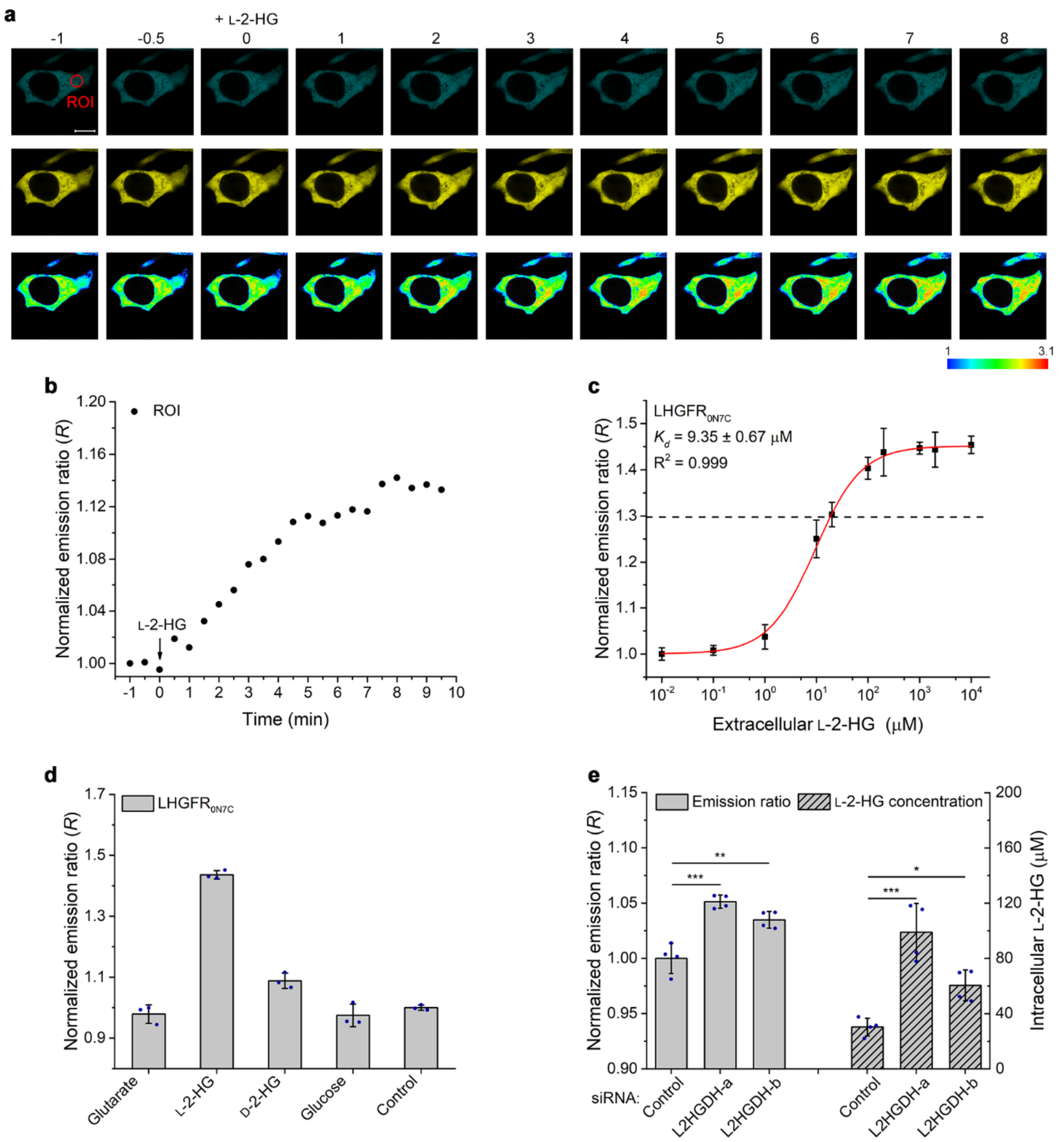

Supplementary Figure 17 Monitoring L-2-HG fluctuations in human cells by LHGFR0N7C. **(a)** Sequential images of mTFP (top), Venus (middle), and Venus/mTFP emission ratio (bottom, pseudocolored) of single HEK293FT cell expressing LHGFR0N7C. 10 mM L-2-HG was added at time point zero (min). Elapsed time (in minutes) after addition of L-2-HG is shown at the top of the images. Scale bar, 10 μm. **(b)** Time course of the emission ratio

changes inside region of interest (ROI) depicted from the top-left image of **(a)**. **(c)** Normalized dose-response curve of $LHGFR_{0N7C}$ expressed in HEK293FT cells with increasing concentrations (10 nM to 10 mM) of L-2-HG. Cells were permeabilized with 10 μM digitonin. The emission ratio of non-permeabilized HEK293FT cells under physiological conditions is indicated with black dash line. **(d)** Responses of $LHGFR_{0N7C}$ expressed in HEK293FT cells to exogenously added 1 mM glutarate, L-2-HG, D-2-HG, and glucose. Cells were permeabilized with 10 μM digitonin. All data were normalized to the control (ratio in the absence of any tested compounds). **(e)** Identification of the function of L2HGDH in L-2-HG catabolism by $LHGFR_{0N7C}$. The emission ratio was measured after co-transfecting siRNA targeting L2HGDH and $LHGFR_{0N7C}$ for 48 h. Emission ratio was normalized to the control condition. *P* values of the emission ratio group were determined as follows: siControl vs. siL2HGDH-a, $P < 0.0001$; siControl vs. siL2HGDH-b, $P = 0.0017$. *P* values of the L-2-HG concentration group were determined as follows: siControl vs. siL2HGDH-a, $P = 0.0002$; siControl vs. siL2HGDH-b, $P = 0.0354$. All data shown are means ± s.d. (n = 3, 3, and 4 independent experiments for **c**, **d**, and **e**). *, $P < 0.05$; **, $P < 0.01$; ***, $P < 0.001$; one-way ANOVA test with Tukey's Multiple Comparison Test. Source data are provided as a Source Data file.

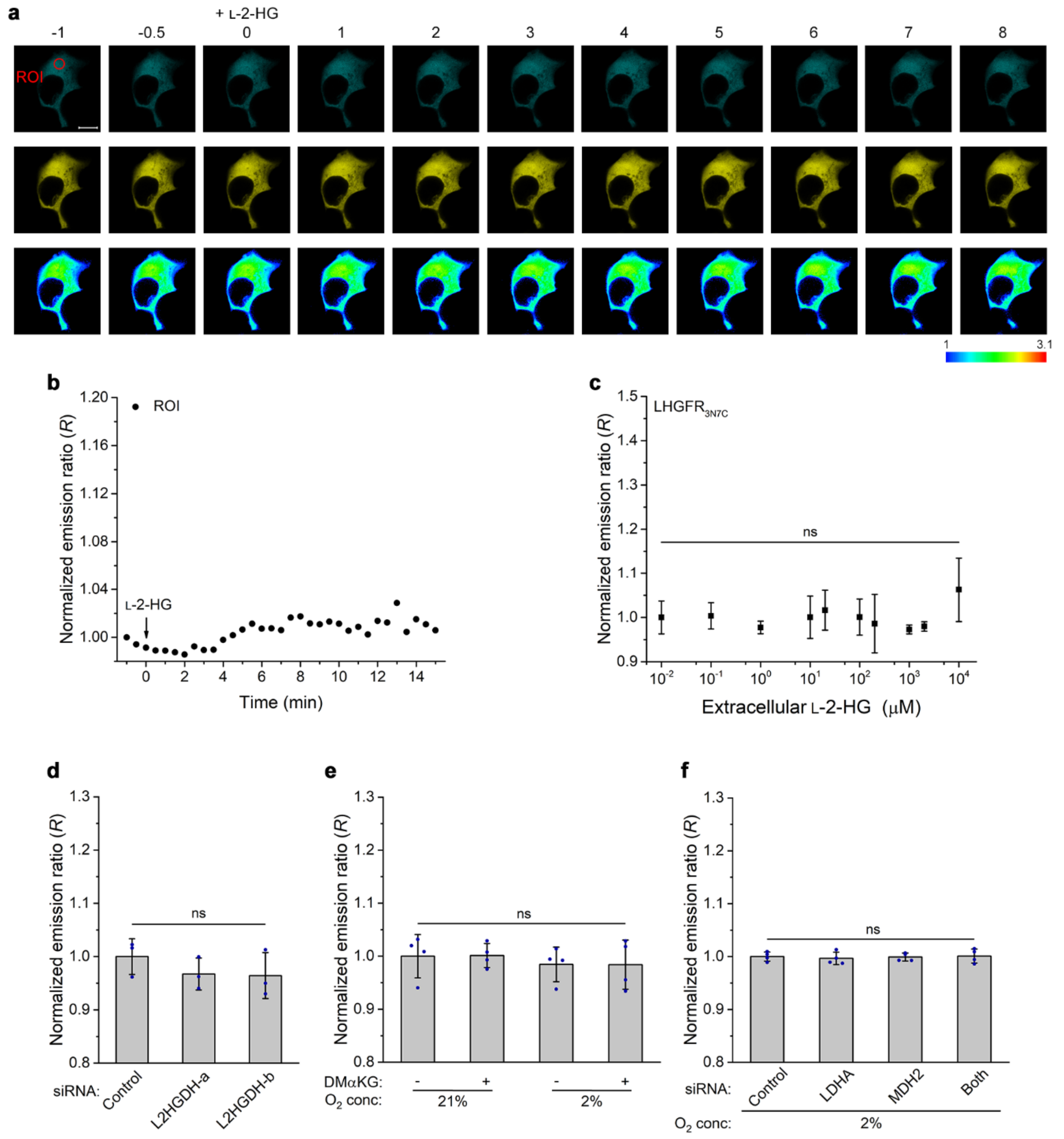

Supplementary Figure 18 Monitoring L-2-HG fluctuations in human cells by LHGFR3N7C. **(a)** Sequential images of mTFP (top), Venus (middle), and Venus/mTFP emission ratio (bottom, pseudocolored) of single HEK293FT cell expressing LHGFR3N7C. 10 mM L-2-HG was added at time point zero (min). Elapsed time (in minutes) after addition of L-2-HG is shown at the top of the images. Scale bar, 10 μm. **(b)** Time course of the emission ratio

changes inside region of interest (ROI) depicted from the top-left image of **(a)**. **(c)** Normalized dose-response curve of $LHGFR_{3N7C}$ expressed in HEK293FT cells with increasing concentrations (10 nM to 10 mM) of L-2-HG. Cells were permeabilized with 10 μM digitonin. **(d)** Identification of the function of L2HGDH in L-2-HG catabolism by $LHGFR_{3N7C}$. The emission ratio was measured after co-transfecting siRNA targeting L2HGDH and $LHGFR_{3N7C}$ for 48 h. **(e)** Detection of hypoxia-induced L-2-HG accumulation by $LHGFR_{3N7C}$. Emission ratio changes were recorded after $LHGFR_{3N7C}$-expressing HEK293FT cells cultured in normoxia or hypoxia in the absence and presence of 5 mM dimethyl-2-ketoglutarate (DMαKG) for 24 h. Emission ratio was normalized to normoxic condition without DMαKG. **(f)** Identification of the functions of LDHA and MDH2 in L-2-HG anabolism by $LHGFR_{3N7C}$. HEK293FT cells were cultured in the presence of 5 mM DMαKG. Emission ratio was normalized to the condition treated with negative siRNA. All data shown are means ± s.d. (n = 3, 3, 4, and 4 independent experiments for **c**, **d**, **e**, and **f**). ns, no significant difference ($P \geq 0.05$); one-way ANOVA test with Tukey's Multiple Comparison Test for **(c)**, **(d)**, and **(f)**; two-tailed *t* test for **(e)**. Source data are provided as a Source Data file.

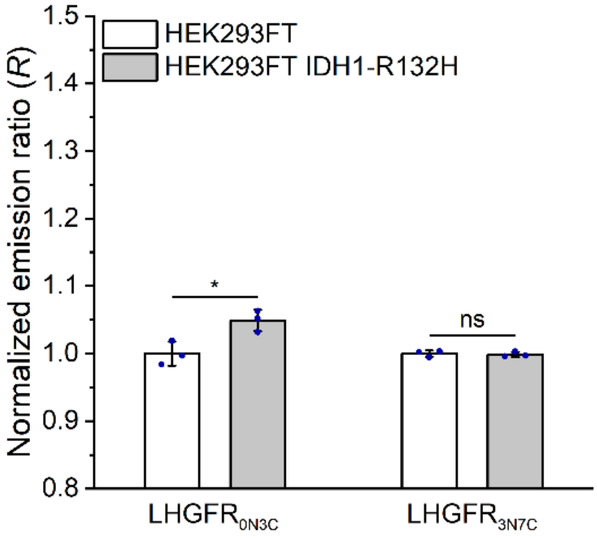

Supplementary Figure 19 The effect of IDH1 mutation-induced D-2-HG accumulation on LHGFR functions. $LHGFR_{0N3C}$ and $LHGFR_{3N7C}$ were expressed in HEK293FT cells with or without IDH1 mutation (IDH1-R132H), and the emission ratios were recorded 48 h following transfection. The data shown are means ± s.d. (n = 3 independent experiments) and normalized to the HEK293FT cells without IDH1 mutation. P = 0.0234, 0.7003 (from left to right). *, $P < 0.05$ in two-tailed t test; ns, no significant difference ($P \geq 0.05$) in two-tailed t test. Source data are provided as a Source Data file.

Supplementary Table 1 LOD, K_d, and ΔR_{max} of LHGFR for L-2-HG and D-2-HG in assay buffer.

		L-2-HG	D-2-HG	L-2-HG in the presence of D-2-HG	L-2-HG in the presence of 2-KG	L-2-HG in the presence of ATP
LHGFR0N3C	LOD (μM)[a]	4.34	872.59	4.99	4.09	3.84
	K_d (μM)[b]	29.33 ± 1.24	ND[d]	32.93 ± 4.60	28.99 ± 1.65	28.05 ± 1.00
	ΔR_{max}[c]	56.13 ± 0.29%	ND[d]	52.13 ± 0.97%	57.30 ± 0.60%	59.21 ± 1.27%
LHGFR0N7C	LOD (μM)[a]	0.70	128.34	1.19	0.76	1.21
	K_d (μM)[b]	7.22 ± 0.38	1,850 ± 910	7.87 ± 0.34	5.95 ± 0.28	5.45 ± 0.26
	ΔR_{max}[c]	60.37 ± 1.30%	ND[d]	33.85 ± 0.71%	57.05 ± 0.65%	57.30 ± 0.51%

[a]The limit of detection (LOD) is calculated by interpolating the average background counts + 3 × standard deviation value. The calculation method was provided by the engineer from PerkinElmer.

[b]K_d indicates apparent dissociation constant.

[c]ΔR_{max} indicates maximum ratio change.

[d]ND indicates the data could not be detected.

Supplementary Table 2 Evaluation of the performance of LHGFR for quantification of L-2-HG in various biological samples.

Condition	Approach	Concentration (μM)			Accuracy (%)[a]			Precision (RSD%)[b]
		Sample 1	Sample 2	Sample 3	Sample 1	Sample 2	Sample 3	
	Standard	8	40	80	100	100	100	
Serum	LC-MS/MS	7.77	45.88	94.85	97.19	114.70	118.56	10.34
	$LHGFR_{0N3C}$	9.11	42.75	94.40	113.88	106.88	117.99	4.98
	$LHGFR_{0N7C}$	8.92	38.19	91.50	111.55	95.48	114.38	9.51
Urine	LC-MS/MS	8.53	38.65	84.62	106.63	96.62	105.77	5.39
	$LHGFR_{0N3C}$	9.49	46.81	89.36	118.62	117.02	111.71	3.12
	$LHGFR_{0N7C}$	8.45	45.77	92.79	105.63	114.42	115.99	4.98
Bacterial culture medium	LC-MS/MS	8.02	50.08	88.33	100.31	125.20	110.41	11.18
	$LHGFR_{0N3C}$	8.04	38.73	79.83	100.46	96.81	99.79	1.96
	$LHGFR_{0N7C}$	8.36	42.28	80.71	104.47	105.69	100.88	2.41

[a]Accuracy% = $\frac{\text{Concentration determined by LC-MS/MS or LHGFR}}{\text{Defined concentration}}$.

[b]Precision% = $\frac{\text{Standard derivation of accuracy}}{\text{Mean value of accuracy}}$.

Supplementary Table 3 LOD and detection range of various methods for quantification of L-2-HG in various biological samples.

Approach	Serum		Urine		Bacterial culture medium	
	LOD (μM)[a]	Detection range (μM)	LOD (μM)[a]	Detection range (μM)	LOD (μM)[a]	Detection range (μM)
HPLC	400	> 400	100	> 100	100	> 100
LC-MS/MS	4	> 4	1	> 1	1	> 1
$LHGFR_{0N3C}$	5.84	5.84 − 4,000	15.74	15.74 − 4,000	5.48	5.48 − 4,000
$LHGFR_{0N7C}$	1.68	1.68 − 400	0.92	0.92 − 400	0.82	0.82 − 400

[a]The limit of detection (LOD) is calculated by interpolating the average background counts + 3 × standard deviation value. The calculation method was provided by the engineer from PerkinElmer.

Supplementary Table 4 LOD and K_d of LHGFR for D-2-HG in various biological samples.

	Serum		Urine		Bacterial culture medium	
	LOD (μM)[a]	K_d (μM)[b]	LOD (μM)[a]	K_d (μM)[b]	LOD (μM)[a]	K_d (μM)[b]
$LHGFR_{0N3C}$	781.90	ND[c]	3,876.40	ND[c]	1,107.24	ND[c]
$LHGFR_{0N7C}$	90.45	ND[c]	159.59	ND[c]	105.87	1,053.45 ± 311.73

[a]The limit of detection (LOD) is calculated by interpolating the average background counts + 3 × standard deviation value. The calculation method was provided by the engineer from PerkinElmer.

[b]K_d indicates apparent dissociation constant.

[c]ND indicates the data could not be detected.
