# Supplementary material for: An l-2-hydroxyglutarate biosensor based on specific transcriptional regulator LhgR
Source: Nat Commun. 2021 Jun 15;12:3619. doi: 10.1038/s41467-021-23723-7 (PMC8206213; doi:10.1038/s41467-021-23723-7)
Supplement: Supplementary file 6 — Description of Additional Supplementary Files [file 41467_2021_23723_MOESM6_ESM.pdf]

Title: Supplementary Movie 1
Description: Imaging of single HEK293FT cell expressing LHGFR0N3C, LHGFR0N7C, or LHGFR3N7C in response to L-2-HG addition. 10 mM L2-HG was added at time point zero (min). Elapsed time (in minutes) after addition of L-2-HG is shown at the bottom right corner.

Title: Supplementary Data 1
Description: Strains and plasmids used in this study.

Title: Supplementary Data 2
Description: Oligonucleotides used in this study.
