# Supplementary material for: An l-2-hydroxyglutarate biosensor based on specific transcriptional regulator LhgR
Source: Nat Commun. 2021 Jun 15;12:3619. doi: 10.1038/s41467-021-23723-7 (PMC8206213; doi:10.1038/s41467-021-23723-7)
Supplement: Supplementary file 7 — Reporting Summary [file 41467_2021_23723_MOESM7_ESM.pdf]

nature research

Corresponding author(s): Chao Gao and Ping Xu

Last updated by author(s): May 11, 2021

Reporting Summary

Nature Research wishes to improve the reproducibility of the work that we publish. This form provides structure for consistency and transparency in reporting. For further information on Nature Research policies, see our Editorial Policies and the Editorial Policy Checklist.

Statistics

For all statistical analyses, confirm that the following items are present in the figure legend, table legend, main text, or Methods section.

n/a	Confirmed	
☐	☒	The exact sample size (n) for each experimental group/condition, given as a discrete number and unit of measurement
☐	☒	A statement on whether measurements were taken from distinct samples or whether the same sample was measured repeatedly
☐	☒	The statistical test(s) used AND whether they are one- or two-sided<br>*Only common tests should be described solely by name; describe more complex techniques in the Methods section.*
☐	☒	A description of all covariates tested
☐	☒	A description of any assumptions or corrections, such as tests of normality and adjustment for multiple comparisons
☐	☒	A full description of the statistical parameters including central tendency (e.g. means) or other basic estimates (e.g. regression coefficient) AND variation (e.g. standard deviation) or associated estimates of uncertainty (e.g. confidence intervals)
☐	☒	For null hypothesis testing, the test statistic (e.g. F, t, r) with confidence intervals, effect sizes, degrees of freedom and P value noted<br>*Give P values as exact values whenever suitable.*
☒	☐	For Bayesian analysis, information on the choice of priors and Markov chain Monte Carlo settings
☒	☐	For hierarchical and complex designs, identification of the appropriate level for tests and full reporting of outcomes
☒	☐	Estimates of effect sizes (e.g. Cohen's d, Pearson's r), indicating how they were calculated

Our web collection on statistics for biologists contains articles on many of the points above.

Software and code

Policy information about availability of computer code

Data collection	The fluorescence intensities were determined by using Kaleido 3.0 (PerkinElmer) and SoftMax Pro Software 7.0.2 (Molecular Devices). Detailed data collection is described in the Methods.
Data analysis	Software for initial data processing was Microsoft Excel 2016, and subsequent analyses were carried out using OriginPro 2016 (OriginLab), OriginPro 2019 (OriginLab), Graphpad Prism 5 (Graphpad), and Graphpad Prism 7 (Graphpad). The imaging data were obtained and processed by Zen 3.1 (Zeiss) and Image-Pro Plus 6.0.

For manuscripts utilizing custom algorithms or software that are central to the research but not yet described in published literature, software must be made available to editors and reviewers. We strongly encourage code deposition in a community repository (e.g. GitHub). See the Nature Research guidelines for submitting code & software for further information.

Data

Policy information about availability of data

All manuscripts must include a data availability statement. This statement should provide the following information, where applicable:

- Accession codes, unique identifiers, or web links for publicly available datasets
- A list of figures that have associated raw data
- A description of any restrictions on data availability

The data supporting the findings of this study are available within the article and its Supplementary Information files and from the corresponding authors on request. Any supplementary information and source data are available in the online version of the paper. A reporting summary for this article is available as Supplementary Information file. Source data are provided with this paper.

Field-specific reporting

Please select the one below that is the best fit for your research. If you are not sure, read the appropriate sections before making your selection.

[x] Life sciences [ ] Behavioural & social sciences [ ] Ecological, evolutionary & environmental sciences

For a reference copy of the document with all sections, see nature.com/documents/nr-reporting-summary-flat.pdf

Life sciences study design

All studies must disclose on these points even when the disclosure is negative.

Sample size	No statistical methods were used to predetermine the sample sizes. Sample sizes were determined based on similar studies to obtain the convincing and compelling results. At least three biological replicates were performed in all experiments, and detailed sample sizes are indicated for each experiment in the manuscript.
Data exclusions	No data was excluded from the analysis.
Replication	All experiments were repeated on at least 3 separate samples using the reported methods. All attempts at replication were successful.
Randomization	Starting material (purified biosensors, bacteria, or human cells) was randomly assigned to different experimental conditions.
Blinding	No blinding was carried out in sensor development and characterizations the sensor in biological samples, bacteria, and human cells. Because 1) all the samples were randomly selected, and each condition was treated identically to eliminate treatment bias; 2) the experimental conditions were obvious for characterizations of sensor and imaging of human cells; and 3) all analyses were performed objectively in the same way by standard experimental procedures.

Reporting for specific materials, systems and methods

We require information from authors about some types of materials, experimental systems and methods used in many studies. Here, indicate whether each material, system or method listed is relevant to your study. If you are not sure if a list item applies to your research, read the appropriate section before selecting a response.

Materials & experimental systems

n/a	Involved in the study	
[x]	[ ]	Antibodies
[ ]	[x]	Eukaryotic cell lines
[x]	[ ]	Palaeontology and archaeology
[x]	[ ]	Animals and other organisms
[x]	[ ]	Human research participants
[x]	[ ]	Clinical data
[x]	[ ]	Dual use research of concern

Methods

n/a	Involved in the study	
[x]	[ ]	ChIP-seq
[x]	[ ]	Flow cytometry
[x]	[ ]	MRI-based neuroimaging

Eukaryotic cell lines

Policy information about cell lines

Cell line source(s)	The cell line used in this work is HEK293FT cell line, which can be purchased from Procell Life Science&Technology (China). No commonly misidentified cell lines were used in the study.
Authentication	Authentication procedures were not used.
Mycoplasma contamination	Cells were not tested for mycoplasma contamination.
Commonly misidentified lines (See ICLAC register)	HEK293FT cells were used, because they are easy to handle, grow rapidly, and can be readily transfected.
